# Characterization of Ultrasonic Bubble Clouds in A Liquid Metal by Synchrotron X-ray High Speed Imaging and Statistical Analysis

**DOI:** 10.3390/ma13010044

**Published:** 2019-12-20

**Authors:** Chuangnan Wang, Thomas Connolley, Iakovos Tzanakis, Dmitry Eskin, Jiawei Mi

**Affiliations:** 1 Department of Engineering, University of Hull, Cottingham Road, Hull HU6 7RX, UK; Chuangnan.wang@ionix.at; 2 Diamond Light Source, Didcot OX11 0DE, Oxfordshire, UK; thomas.connolley@diamond.ac.uk; 3 Oxford Brooks University, Oxford OX33 1HX, Oxfordshire, UK; itzanakis@brookes.ac.uk; 4 Brunel Centre for Advanced Solidification Technology, Brunel University London, Uxbridge UB8 3PH, UK; Dmitry.Eskin@brunel.ac.uk

**Keywords:** ultrasonic bubble clouds, synchrotron X-ray imaging, metal solidification, ultrasound melt processing

## Abstract

Quantitative understanding of the interactions of ultrasonic waves with liquid and solidifying metals is essential for developing optimal processing strategies for ultrasound processing of metal alloys in the solidification processes. In this research, we used the synchrotron X-ray high-speed imaging facility at Beamline I12 of the Diamond Light Source, UK to study the dynamics of ultrasonic bubbles in a liquid Sn-30wt%Cu alloy. A new method based on the X-ray attenuation for a white X-ray beam was developed to extract quantitative information about the bubble clouds in the chaotic and quasi-static cavitation regions. Statistical analyses were made on the bubble size distribution, and velocity distribution. Such rich statistical data provide more quantitative information about the characteristics of ultrasonic bubble clouds and cavitation in opaque, high-temperature liquid metals.

## 1. Introduction

Ultrasonic cavitation created by high-power ultrasound in liquids is a highly dynamic and nonlinear process. It has very wide applications in industry, for example, ultrasound cleaning [1], sonochemistry [2], metallurgy [3,4]. Historically, cavitation was first studied by Lord Rayleigh in the late 19th century, when he considered the collapse of a spherical void within a liquid [4,5]. During and after the 2nd World War, cavitation in water was studied extensively in the field of hydrodynamics, because it was a very common phenomenon that significantly affected the efficiency of pumps and propellers used for ships [6,7]. For ultrasonic cavitation, many invasive and non-invasive experimental methods have been developed for measuring and characterizing ultrasonic cavitation zone and its intensity. For example, acoustic hydrophones, cavitation meters and foil testing are typical invasive techniques. The probe/sensor or testing foil is inserted into the ultrasonic cavitation region to measure the characteristics of the cavitation zone, its size, position and intensity [8,9,10]. Non-invasive techniques include ultrasonic phase array transducers with ultrasonic imaging, visible light imaging [8,11,12] and Optical Diffraction Tomography (ODT) [13,14]. In non-invasive measurement techniques, no probes are used to interfere with the acoustic/cavitation field.

Photography is a common method for measuring ultrasound cavitation in light transparent liquids. For example, luminol mapping can measure cavitation intensity distribution directly [15,16] based on the brilliance of the emitted light in the cavitation process. ODT uses the diffraction of light by ultrasound and a tomographic technique to form images for the pressure in a plane perpendicular to the ultrasound propagation direction. However, to study the highly dynamic behavior of cavitation, higher temporal and spatial resolution are needed. Hence, high-speed cameras with a higher magnification objective lens is often used [8,17]. For example, Geisler studied bubble oscillation using an acquisition rate of 2 million frames per second in a view field of 160 µm × 160 µm. While Ohl investigated bubble collapse in water using an exposure time of 48 ns, but a single-shot image in a view field of 1.5 mm × 1.8 mm [8,17]. These high-speed imaging studies resulted in significant progress on the understanding of bubble oscillation, bubble shock wave emission, bubble luminescence and liquid flow in the vicinity of bubbles. However, in the cases of opaque, high-viscosity and high-temperature materials such as liquid metals, the methods described above are not suitable.

An in-depth understanding of the interactions of ultrasonic waves with liquid and solidifying metals is essential for developing optimal processing strategies for ultrasound processing of metal alloys [4,18,19]. Recently, researchers in Mi’s group have extensively studied the dynamics of ultrasonic bubbles in liquid metals and their interactions with the growing phases in the liquid, and with the solid-liquid interface [20,21,22,23,24]. These studies have provided real-time and convincing evidence to clarify that (1) the shock wave created at bubble implosion, and (2) the cyclic fatigue effects due to bubble oscillation are the most important mechanisms for the fragmentation of growing dendritic structures and intermetallic phases during the metal solidification processes. High-power ultrasound normally produces a large amount of bubbles in liquid metals (often called a bubble cloud). So far, a quantitative characterisation of those bubble clouds in liquid metals have not been reported. In this aspect, real-time and quantitative studies of ultrasonic bubble clouds in liquid metal can provide more in-depth understanding of the characteristics of ultrasonic bubble clouds.

## 2. Experiments

### 2.1. Alloy and Sample Preparation

**Figure 1:**
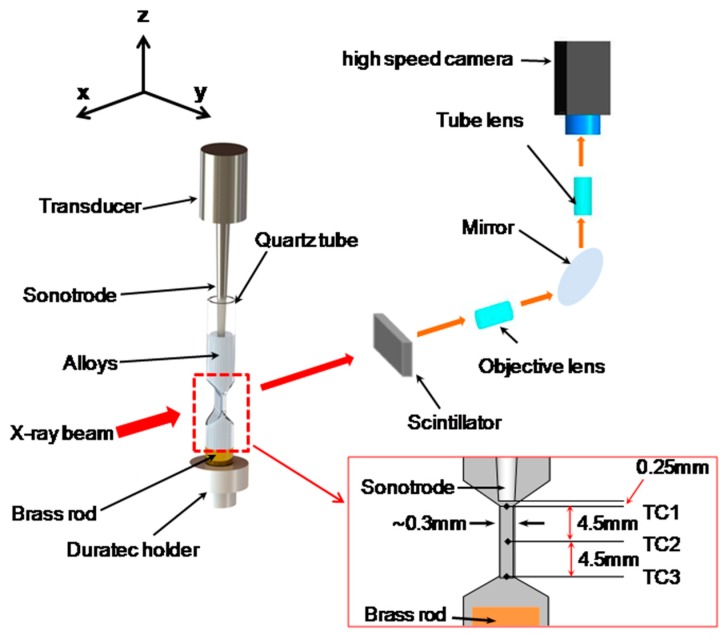
A 3D CAD rendition, showing the experimental set-up; and a 2D sketch of the structure of sample holder, sonotrode and thermal couple (TC 1, 2 and 3) position.

A Sn-30wt%Cu alloy was chosen as the experimental alloy. It has a wide solidification range, allowing ultrasound to be applied over a wide range of temperatures (from 250 to 750 °C). Furthermore, the Sn–Cu binary system [25] is the key alloy system for lead-free soldering materials, and intermetallic compounds such as Cu_6_Sn_5_ are promising candidates for enhancing the storage capacity of Lithium ion-based batteries. Samples were contained inside specially made quartz capsules with a flattened “hourglass” shape, as shown in Figure 1. The thin central window section of the capsule was 300 µm thick to allow good X-ray penetration. The sonotrode was positioned in the upper end of the hourglass, with the tip close to the top of the thin section. A heat sink made of a stainless steel rod was placed at the bottom reservoir of the hourglass capsule to create a thermal gradient. The alloy in the capsule was melted inside a small cartridge heater furnace [26]. Sample temperature was monitored and recorded by three K-type thermocouples placed at the top, middle and bottom of the thin window section. The details of the experiment are very similar to those described in [22,24].

### 2.2. High-Speed Synchrotron X-ray Imaging

The experiment was carried out at Beamline I12, Diamond Light Source, Oxford, UK [27], with the set up illustrated in Figure 1.

I12 has a wiggler source that delivers a peak flux of ~1.7 × 10^11^ photons/s/mm^2^/0.1%BW in the first experimental hutch. Filtered white beam was used to give maximum X-ray flux on the sample for high-speed image acquisition. Filtration of the beam (4 mm of copper) was necessary to reduce heat load on the sample and reduce the risk of damage to the X-ray detector. The X-ray camera system consisted of a 200 µm thick LYSO scintillator lens coupled to a Miro 310M high-speed CMOS camera operating at a 1280 × 800 pixel image size with a resolution of 4 µm × 4 µm per pixel. Under these conditions with the given samples, frame rates of 2000 frames per second (fps) were obtained, using an exposure of 499 μs, for a total recording time of 4 s. The recording time was limited by the camera’s fast onboard memory buffer (12GB). However, this was adequate for observing the ultrasonic phenomena in the experiments. Flat field images without a sample were recorded to enable correction for non-uniformity in the incident beam intensity profile and imperfections in the imaging system.

Once the desired target temperature (675 °C) was reached and stabilized in the liquid metal, a TTL trigger unit was used to initiate recording of images using a high-speed X-ray camera. After a ~10 ms delay, ultrasound was applied to the liquid metal. The ultrasound was generated using an UP100H ultrasonic processor with an MS2 sonotrode (Hielscher ultrasound technology Ltd., Teltow, Germany). Ultrasound powers of 20 W, 60 W and 100 W were used in the experiment to create bubble clouds with different characteristics. In this way, the whole process of ultrasound bubble nucleation, growth and propagation can be captured.

Two X-ray videos were taken for each ultrasonic power setting. The first video was taken of the area just below the sonotrode tip, with the aim of studying cavitation phenomena close to the tip. In this paper, the location is referred to as chaotic cavitation region (CCR). A second video was taken 1.5 mm below the first set of images. This second video was targeted at phenomena which happen further away from the sonotrode tip, referred to as the quasi static cavitation region (SCR) in this paper.

## 3. Image Processing and Data Analysis

**Figure 2:**
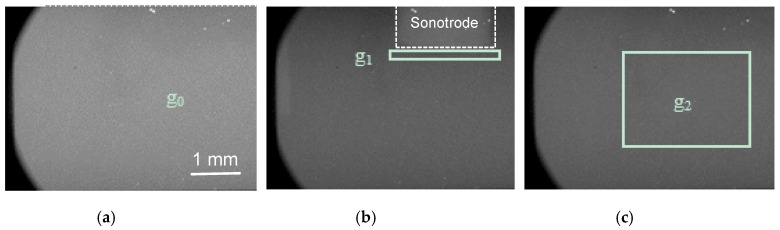
X-ray images of liquid Sn-30wt%Cu alloy, without the application of ultrasound, (**a**) a typical flat-field image, and the black regions at the corners of the images are from the lens mount of the camera. (**b**) in the chaotic cavitation region with the sonotrode (marked with the white dotted line) in the view field; (**c**) in the quasi static cavitation region, i.e., 1.5 mm below the sonotrode. *g*_0_, *g*_1_ and *g*_2_ mark the locations (including the grey level) where the thicknesses of the liquid metal are known as listed in Table 1.

Flat-field images were taken without any sample between the X-ray source and detector, as shown in Figure 2a. The images were then filtered using the Remove Outlier function in ImageJ [28] to remove random bright outlier pixels caused by direct X-ray strikes on the camera sensor. Such strikes are the consequence of the high-intensity, high-energy beam and cannot totally be eliminated by shielding of the camera system. The acquired videos were flat-field corrected by dividing the flat-field image to remove the effect of non-uniform X-ray beam intensity and compensate for systematic variations in the detector system, such as vignetting, or dust or scratches on the scintillator. Dark count and bad pixel correction were also performed by the camera hardware. Typical images obtained after flat-field correction and filtering are presented in Figure 2b,c.

In the experiments, high-speed images sequences in the CCR and SCR regions were captured under different ultrasound powers. In the CCR, the ultrasonic bubbles are highly dynamic and interconnected. In the SCR, individual ultrasonic bubbles were clearly seen. As bubbles attenuate much less X-rays than the liquid Sn-30wt%Cu alloy, they had a higher grey level in images than the surrounding liquid alloy and can be segmented, and individually counted and measured. To quantify the different cavitation characteristics, different methods were used. Cavitation in the CCR was analysed based on grey-level differences, while cavitation in the SCR was analysed by counting and measuring individual bubbles.

### 3.1. Bubble Volume Fraction in the Chaotic Cavitation Region

In the CCR region, ultrasonic bubbles were highly dynamic and interconnected to form bubble clouds. It was impossible, at 2000 fps, to capture and distinguish the individual bubbles. To characterise the collective behaviour of the bubble clouds, the acquired images were analysed based on the grey-level distribution caused by the presence of the bubble clouds, because they have lower X-ray attenuation, and result in a brighter area in the images. If monochromatic (single-wavelength) X-rays were used, the transmitted intensity through the sample can be calculated analytically from a known X-ray mass attenuation coefficient *μ* using the Beer–Lambert Law [29]. For an incident intensity *I_o_*, the transmitted intensity *I* through a material of density *ρ* and thickness *d* is given by:

(1) $$I=I_{o}e^{-\mu \rho d}$$

*μ* is a material property and it is a function of photon energy. However, in this study, monochromatic X-rays do not have sufficient flux to achieve the short exposure times associated with the high frame rate that is required in this experiment. Instead, broad spectrum multi-wavelength X-rays, known as white-beam X-rays, were used. For white-beam X-rays, the Beer–Lambert exponential attenuation law cannot be used directly, because both the mass attenuation coefficient of the sample and the X-ray detector response are photon-energy-dependent. Therefore, an approximation method based on grey-level measurement is adopted to calculate the bubble cloud volume fraction in this paper. The diameter of the sonotrode tip was 2 mm, which means that the thickness of the material just under the sonotrode tip, indicated by the rectangle in Figure 2b, was approximately 2 mm. As showed in the insert of Figure 1, the narrow tube thickness was 0.3 mm (the corresponding X-ray image through this area is illustrated in Figure 2c). The thicknesses at these two positions were used as the reference to calculate the overall attenuation coefficients. We assumed that the sample thickness and grey level are exponentially related. At both positions, the empirical attenuation coefficients were approximated by the equation below:

(2) $$g=g_{0}e^{-\alpha_{g}x+\beta_{g}}$$

Here, *g*_0_ is the grey level of the corresponding position in the flat-field image, *x* is the transmitting distance and *α_g_* and *β_g_* are the empirical attenuation coefficients, representing the attenuation parameters in this case. Using the grey levels found at the locations of *g*_0_, *g*_1_ and *g*_2_ (see Table 1), *α_g_* and *β_g_* are calculated as 124.2 and −0.33, respectively. Equation (2) was then used to calculate the thickness of the liquid Sn-30wt%Cu (i.e., *x* in Equation (2)). The line density along the X-ray propagation direction where ultrasonic bubbles were found can be calculated by using:

(3) $$LD=(D_{0}-D)/D_{0}$$

where *D*_0_ is the thickness of the liquid metal without ultrasonic bubbles, and *D* is the thickness of the sample containing ultrasonic bubbles. Both are calculated by Equation (2). For a unit volume, (a unit area perpendicular to the X-ray transmission direction times a unit length along the X-ray direction), the volume fractions of the bubbles in this unit volume at the CCR can be calculated using Equation (3) and are actually represented by LD.

### 3.2. Bubble Volume Fraction in the Quasi Static Cavitation Region

**Figure 3:**
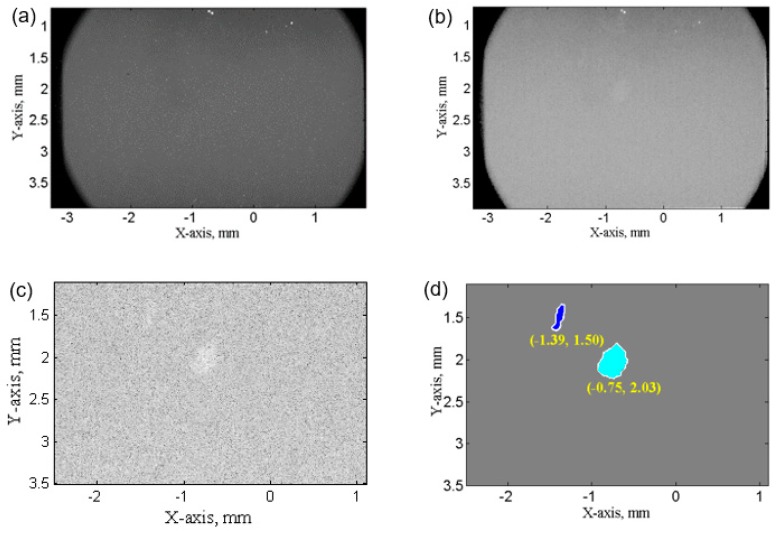
A typical X-ray raw image taken in the SCR region. (**a**) before image processing; (**b**) after flat-field correction; (**c**) cropped X-ray image after normalization by image taken without UST, and contrast enhancement; (**d**) The final binary image, which was used to extract the position and size of ultrasonic bubbles.

For the SCR region (1.5 mm below the region that contains the sonotrode), individual ultrasonic bubbles or bubble clouds were observed and recorded. To count these bubbles, the images were firstly normalized by the X-ray images that were taken without ultrasound treatment (UST) to remove any non-uniform grey level caused by the non-uniform thickness of the glass cell windows. Secondly, the contrast between bubbles and background was enhanced by using the inverse of a natural exponential function as demonstrated in Figure 3c. Thirdly, the image was converted into binary images based on the predefined grey-level threshold. Finally, the bubble dimensions and positions were determined using the MATLAB Image Processing Toolbox, which is illustrated in Figure 3d. In this way, a statistical analysis of ultrasonic bubbles was made and described in Section 4.

## 4. Experimental Results

### 4.1. Bubble Cloud in the Chaotic Cavitation Region 

**Figure 4:**
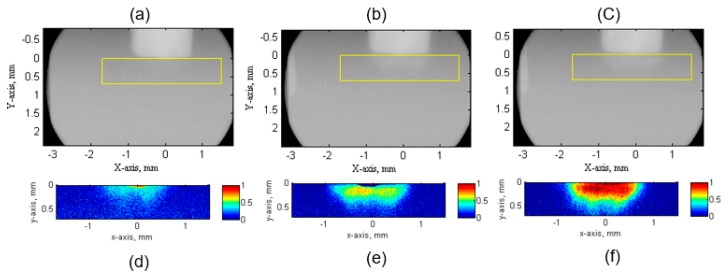
High-speed synchrotron X-ray images of ultrasonic bubble clouds near the sonotrode tip at different ultrasound powers of (**a**) 20 W, (**b**) 60 W, (**c**) 100 W respectively. (**d**–**f**) show the corresponding bubble volume fraction calculated based on Equation (3).

The ultrasound bubble clouds observed in the CCR, at different ultrasound powers (20 W, 60 W and 100 W, respectively) are illustrated in Figure 4. In each case, 10 sequential X-ray images were averaged to reduce background noise, so that each result in the figure contained the information averaged over 5 ms. Figure 4d–f, shows the bubble volume fraction calculated using Equation (3).

**Figure 5:**
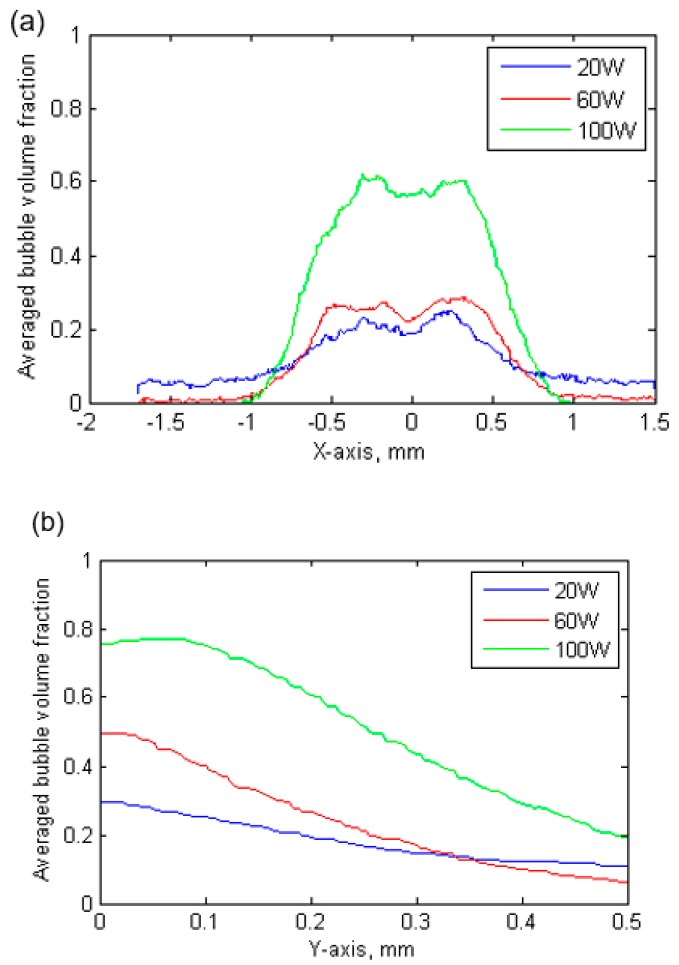
The averaged bubble volume fraction in (**a**) horizontal direction from position −1.5 mm to 1.5 mm (*X*-axis), to show bubble volume fraction distribution in horizontal direction; in (**b**) vertical direction from sonotrode tip to 0.5 mm (*Y*-axis), to show bubble volume fraction distribution in vertical direction.

Figure 4d–f clearly show that the bubble volume fraction increases with increasing ultrasound power. Figure 5 further plots the line distribution characteristics in the horizontal and vertical directions. Figure 5b clearly shows that the maximum cavitation occurred near the sonotrode tip. Measurements of cavitation intensity and cavitation cloud dimensions are summarized in Table 2. Cavitation cloud width and length in the x and y direction are characterised by the full width at half maximum (FWHM). The measurement indicated that there was no significant variation in the physical dimensions of the cavitation cloud. However, cavitation intensity, which was evaluated in terms of the average bubble volume fraction, increased significantly with the increase of ultrasound power.

### 4.2. Bubble Cloud in the Quasi-Static Cavitation Region

**Figure 6:**
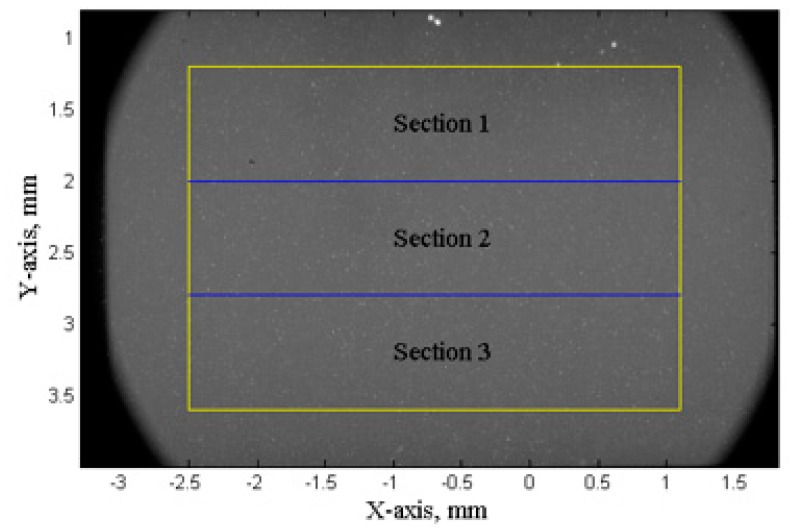
A typical X-ray image of the SCR and three sub-regions where data analysis was made.

In the SCR, individual bubbles were observed and hence tracking and counting of the imaged bubbles are possible. MATLAB was employed to track and measure bubble sizes and velocities. The region of interest for the measurement is shown by the yellow box in Figure 6. The region of interest was further divided into three sub regions: Section 1 (1.2 mm to 2 mm, distance to sonotrode), Section 2 (2 mm to 2.8 mm, distance to sonotrode), Section 3 (2.8 mm to 3.6 mm, distance to sonotrode). A statistical study of bubbles was performed on a sequence of 2000 X-ray images (1 s duration of ultrasound processing) acquired in a quasi steady-state condition (1 s after the ultrasound was turned on, and a steady-state condition was established in the liquid alloy).

#### 4.2.1. Bubble Size Distribution

**Figure 7:**
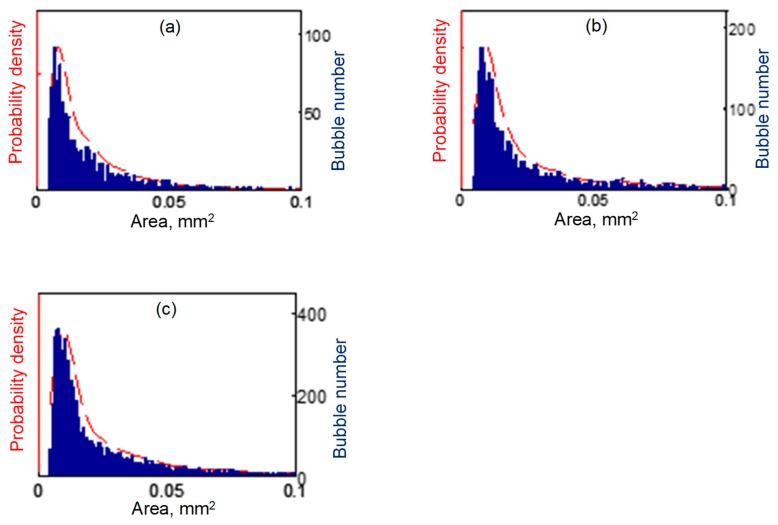
Ultrasonic bubble sizes distribution in the SCR with ultrasound power of (**a**) 20 W, (**b**) 60 W and (**c**) 100 W respectively.

Figure 7 shows the bubble size distribution for different ultrasound powers. The Kernel probability density function [30] was used to calculate the probability densities of these bubble size distributions. The maximum probability density occurred at a bubble size of 0.0078 mm^2^, 0.0098 mm^2^ and 0.0096 mm^2^ (in Table 3) for 20 W, 60 W and 100 W ultrasonic powers, respectively. These results indicate that bubbles with a diameter of approximately 0.01 mm^2^ were dominant in the bubble population. The observed bubble size was mainly dependent on two factors: (1) the initial bubble radius (i.e., the nuclei size), which is related to the liquid properties (viscosity, density, etc.) and (2) ultrasound pressure magnitude and frequency. It should note that the camera resolution is 4 µm per pixel, which means that a bubble diameter smaller than 10 µm cannot be resolved.

The statistical results show that 20 W ultrasound power generated 16 bubbles with an area larger than 0.1 mm^2^, which is 1.6% of its total bubble population. A 60 W ultrasound power generated 234 bubbles with an area larger than 0.1 mm^2^, which is 9.1% of its total bubble population; a 100 W ultrasound power generated 1151 bubbles with an area larger than 0.1 mm^2^, which is 17.5% of its total bubble population. Clearly, a higher ultrasound power resulted in a larger probability of bigger-sized bubbles.

#### 4.2.2. Bubble Velocity Distribution 

**Figure 8:**
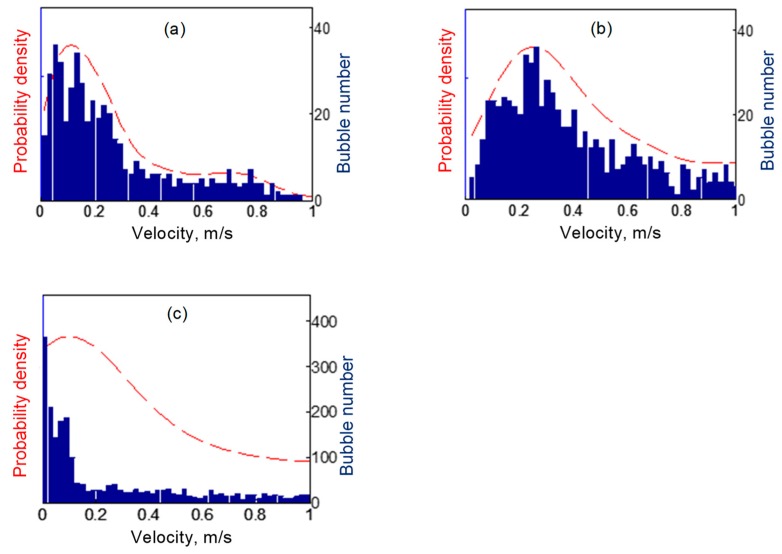
Bubble velocity distributions in the SCR at ultrasound powers of (**a**) 20 W, (**b**) 60 W, (**c**) 100 W, respectively.

Individual bubble velocities were calculated by comparing the position of a bubble centroid in consecutive images. The distances between the centroids of the same bubble in consecutive frames were taken as the distance travelled in the interframe time of the image sequence. The velocities of bubbles were calculated by dividing the measured distance during the interframe time. By measuring the bubble velocities in a one second image sequence, velocity distributions were obtained and are plotted as histograms in Figure 8 with the probability densities plotted as dashed lines. The velocities corresponding to the maximum probability density were 0.15 m/s, 0.28 m/s and 0.17 m/s, for 20 W, 60 W and 100 W ultrasonic powers, respectively. The most probable velocity at 100 W (0.17 m/s) was lower than that at 60 W. We think this may be due to the imaging method we used. At 100 W power, some bubble velocities were too fast for the same bubble to be captured in two consecutive images. This means that some fast-moving bubbles are missing from the bubble velocity datasets, and statistical analysis was only performed on bubbles that were moving slowly enough to be captured in two consecutive frames.

Imaging at higher frame rates would be able to measure the velocity of fast-moving bubbles in the 100 W case. However, with the given camera sensitivity, sample and X-ray source characteristics, the signal/noise ratio in even higher-speed images would be lower, making bubble identification more difficult. 

### 4.3. Limitation of the Imaging Method

In this research, we acquired many terabytes of image sequences. To extract useful information from these large-scale image datasets, a simple but robust method had to be developed. In our case, ultrasound bubbles away from the sonotrode, which we refer it as SCR, were identified based on the grey level. It is interesting to see that, in the field of view of the images taken in this research, the bubble size distribution away from the sonotrode does not change significantly as the ultrasound power increases, although normally, a higher acoustic pressure would lead to larger bubbles. For characterising the bubbles, the imaging method was limited by the acquisition frame rate (2000 fps), especially for the case of 100 W, where bubbles can travel out of the view field in two consecutive images, making the tracking of the exact location of the bubble more difficult.

In our experiment, the ultrasound cavitation bubble cloud is found to be in a relative stable shape, and it is due to the higher ultrasound attenuation of the liquid metal.

## 5. Conclusions

Synchrotron X-ray high-speed imaging was used to study the aggregate behaviour of ultrasonic cavitation bubbles, i.e., bubble cloud in a liquid alloy Sn-30%wtCu. A new method based on the X-ray attenuation for a white X-ray beam was developed to extract the quantitative information about the bubble clouds in the chaotic and quasi-static cavitation regions. This method is generic and applicable to all liquid metals. Statistical analyses were made on the bubble size distribution, and velocity distribution. Such rich statistical data provide more quantitative information about the characteristics of ultrasonic bubble clouds and cavitation in opaque, high-temperature liquid metals.

## Figures and Tables

**Table 1:** Grey level and thickness of the three locations, *g*_0_, *g*_1_ and *g*_2_ shown in Figure 2.

Position	Thickness of Liquid Metal (mm)	Grey Level
*g*_0_	0	145
*g*_1_	2	89
*g*_2_	0.3	97

**Table 2:** Ultrasonic bubble cloud characteristics due to different ultrasonic powers.

Ultrasound Power (W)	Ultrasonic Bubble Cloud * FWHM in *X*-axis (mm)	Ultrasonic Bubble Cloud FWHM in *Y*-axis (mm)	Maximum Averaged Bubble Volume Fraction in *X*-axis	Maximum Averaged Bubble Volume Fraction in *Y*-axis
20	1.15	0.30	0.22	0.31
60	1.26	0.30	0.38	0.52
100	1.26	0.34	0.71	0.78

* FWHM is the full width at half maximum.

**Table 3:** Ultrasonic bubble size and velocity distribution.

Ultrasound Power (W)	Bubble Size at Maximum Probability Density (mm^2^)	Bubble Velocity at Maximum Probability Density (m/s)
20	0.0078	0.15 m/s
60	0.0098	0.28 m/s
100	0.0096	0.17 m/s
